# Development and clinical trial of M701, an Anti-EpCAM × Anti-CD3 bispecific antibody: a targeted intraperitoneal therapy for malignant ascites stemming from advanced solid tumors

**DOI:** 10.1186/s40164-025-00727-3

**Published:** 2025-11-22

**Authors:** Rongrui Liu, Rongbo Lin, Ning Li, Guiling Li, Tao Zhang, Jun Zhao, Jiayi Li, Meili Sun, Ke Wang, Hanxiang An, Weijie Zhang, Huiting Xu, Shan Zeng, Mingjun Zhang, Wei Duan, Yuxian Bai, Jingdong Zhang, He Tian, Fei Yin, Yu Kang, Qi Xu, Nong Xu, Yanhong Deng, Qing Chen, Yongqiang Li, Hongying Yang, Fang Su, Zhenghong Xiao, Xiaojun Xiang, Pengfei Zhou, Shaoyi Huang, Jing Zhang, Jianming Xu

**Affiliations:** 1 Department of Oncology, The First Medical Center, Chinese PLA General Hospital, 28 Fuxing Road, Beijing, 100039 China; 2 Department of Abdominal Oncology, Fujian Cancer Hospital, No. 420 Fuma Road, Fuzhou, 350000 China; 3 Department of Gastrointestinal Oncology, The Affiliated Cancer Hospital of Zhengzhou University & Henan Cancer Hospital, No. 127 Dongming Road, Zhengzhou, 450000 Henan China; 4 Department of Gynecologic Oncology, Cancer Center of Union Hospital, Tongji Medical College, Huazhong University of Science and Technology, No. 1277 Jiefang Avenue, Wuhan, 430000 Hubei China; 5 Department of Abdominal Oncology, Cancer Center of Union Hospital, Tongji Medical College, Huazhong University of Science and Technology, No. 1277 Jiefang Avenue, Wuhan, 430000 China; 6 Department of Oncology, Changzhi People’s Hospital, The Affiliated Hospital of Changzhi Medical College, No. 502 Changxing Middle Road, Changzhi, 046000 China; 7 Department of Medical Oncology, School of Medicine, The First Affiliated Hospital of Xiamen University, Xiamen University, No. 55 Zhenhai Road, Xiamen, 361005 China; 8 Department of Oncology, Jinan Central Hospital affiliated to Shandong University, No. 105 Jiefang Road, Jinan, 250013 China; 9 Department of Gynecological Oncology, Tianjin Medical University Cancer Institute and Hospital, West Huan-Hu Road, Tianjin, 300060 China; 10 Department of Gastrointestinal Oncology, The Cancer Center, Shanxi Bethune Hospital, Shanxi Medical University, No. 99 Longcheng Street, Taiyuan, 030032 China; 11 Department of Oncology, The First Affiliated Hospital of Zhengzhou University, No. 1 Jianshe Dong Road, Zhengzhou, 450052 China; 12 Department of Medical Oncology, Hubei Cancer Hospital, No. 116 Zhuodaoquan South Road, Wuhan, 430079 China; 13 Department of Chemotherapy Oncology, Xiangya Hospital of Central South University, No. 87 Xiangya Road, Changsha, 410008 China; 14 Department of Oncology, The second hospital of Anhui medical university, No. 678 Furong Road, Hefei, 230601 China; 15 Department of Gynecologic Oncology, Beijing Obstetrics and Gynecology Hospital, Capital Medical University, No. 17 Qihelou, Beijing, 100069 China; 16 Department of Gastrointestinal Medical Oncology, Harbin Medical University Cancer Hospital, 150 Haping Road, Harbin, 150040 China; 17 Department of Gastroenterology, Liaoning Cancer Hospital & Institute, No. 44 Xiaoheyan Road, Shenyang, 110042 China; 18 Department of Gastroenterology, Shandong Cancer Hospital, No. 440 Jiyan Road, Jinan, 250117 China; 19 Department of Gastroenterology, The Fourth Hospital of Hebei Medical University, No. 12 Jiankan Road, Shijiazhuang, 050011 China; 20 Department of Gynecological Oncology, Obstetrics and Gynecology Hospital of Fudan University, No. 419 Fangxie Road, Shanghai, 200011 China; 21 Department of Hepato-Pancreato-Biliary & Gastric Medical Oncology, Zhejiang Cancer Hospital, No. 1 East Banshan Road, Hangzhou, 310022 China; 22 Department of Oncology, The First Affiliated Hospital, Zhejiang University School of Medicine, No. 79 Qingchun Road, Hangzhou, 310003 China; 23 Department of Oncology, The Sixth Affiliated Hospital, Sun Yat-sen University, No. 26 Yuancun Er Heng Road, Guangzhou, 510655 China; 24 Department of Obstetrics and Gynecology, Sun Yat-sen Memorial Hospital, Sun Yat-sen University, No. 107 Yanjiang Road West, Guangzhou, 510120 China; 25 The First Department of Chemotherapy, Affiliated Cancer Hospital of Guangxi Medical University, 71 Hedi Road, Nanning, 530021 China; 26 Department of Gynaecology, Yunnan Cancer Hospital, No. 519 Kunzhou Road, Kunming, 650118 China; 27 Department of Oncology, The First Affiliated Hospital of Bengbu Medical University, 287 Changhuai Road, Bengbu, China; 28 Department of Oncology, Nanshi Hospital of Nanyang, No. 130 Zhongzhou West Road, Nanyang, 473001 China; 29 Department of Oncology, The First Affiliated Hospital of Nanchang University, No. 17 Yongwai Main Street, Nanchang, 330006 China; 30 Wuhan YZY Biopharma Co. Ltd., C2-1 Building, Guanggu Biolake, No. 666, Gaoxin Avenue, Wuhan, 430000 China; 31 Department of Oncology, The Fifth Medical Center, Chinese PLA General Hospital, No. 8 Dongdajie, Beijing, 100071 China

**Keywords:** EpCAM, CD3, Bispecific antibody, Malignant ascites, Intraperitoneal therapy, Puncture-free survival

## Abstract

**Background:**

Malignant ascites (MA) is one of the major complications in advanced epithelial cancer patients and is associated with poor prognosis, poor quality of life, and severe symptoms. No efficient medicine is available for treating MA worldwide. Only paracentesis is recommended by the guidelines in most countries, but with limited efficacy and a short control time. Thus, novel treatments are needed to control MA.

**Methods:**

An anti-EpCAM × anti-CD3 bispecific antibody, M701, was constructed as a T-cell engager to eliminate tumor cells in the peritoneal cavity. A phase II study was performed to evaluate the efficacy and safety of the intraperitoneal (IP) infusion of M701 in advanced epithelial tumor patients with moderate-to-large-scale MA. In this study, 84 patients were enrolled, with 43 in the M701 group receiving paracentesis and IP M701 infusion and 41 in the control group receiving paracentesis alone.

**Results:**

The primary endpoint, median puncture-free survival (PuFS), was 75 days in the M701 group and 25 days in the control group, with a significant difference (*p* = 0.0065). Subgroup analysis indicated that different types of cancer, including gastric, colorectal, and ovarian cancers, all benefited from the M701 infusion. Patients with higher relative lymphocyte counts (≥ 13%) at baseline received better effects. Compared to patients in the control group, the overall survival (OS) of patients in the M701 group was certain extended (mOS 110 days vs. 76 days, *p* = 0.1443, HR = 0.68). The 6-month survival rates were 33.3% and 12.1% in the two groups, respectively. No additional serious adverse events (SAEs) were detected in the M701 group. The most frequent treatment-related adverse events were anemia and low white blood cell count, which were manageable. M701 infusions did not cause a greater risk than paracentesis alone in the control arm, while all patients were administered systemic treatment.

**Conclusion:**

When treated with M701, patients with MA had significantly longer puncture intervals and a trend of extended survival time. The results were encouraging for patients with MA. A phase III clinical trial of M701 aimed at further validation is ongoing.

**Supplementary Information:**

The online version contains supplementary material available at 10.1186/s40164-025-00727-3.

## Background

Malignant Ascites (MA) refers to the accumulation of fluid in the abdominal cavity due to the presence of various malignant tumors and is most commonly associated with gastrointestinal and gynecological cancers [1–3]. The presence of MA indicates that the primary tumor has metastasized either locally or systemically. The build-up of moderate-to-large volumes of fluid often compresses nearby organs, impairing their function. It can also lead to symptoms such as abdominal pain, breathing difficulty, electrolyte imbalances, hypoproteinemia, and secondary infections [3, 4]. The prognosis is dire, and the disease imposes a heavy burden on patients, significantly worsening their quality of life [3–5].

No globally approved drug is available that specifically targets the treatment of MA, and no established guidelines or consensus on its diagnosis and management are available [1, 4, 5]. Clinically, the primary method of symptom relief involves paracentesis or catheter drainage, which offers immediate alleviation. These procedures are often supplemented by treatments, such as diuretics, or the IP administration of chemotherapy or antiangiogenic agents, based on clinical experience. However, these approaches lack robust evidence from clinical trials and have limitations regarding safety and efficacy [5–7].

Epithelial cell adhesion molecule (EpCAM) is a transmembrane glycoprotein that functions as an adhesion molecule [8–10]. The 39–42 kDa protein consists of a large extracellular domain with two epidermal growth factor-like repeats, a single transmembrane region, and a short cytoplasmic tail of 26 amino acids [11]. It is extensively expressed in normal epithelial tissues, including gastric, colon, pancreas, ovarian, and lung tissues, and has low expression levels [12, 13]. It is frequently overexpressed in various epithelial malignancies in patients and is associated with poor prognosis [14–18]. Treatment of head and neck cancer cells, as well as breast cancer cells, with EpCAM-specific antisense oligonucleotides or siRNAs has led to significant reductions, or even complete inhibition, of cell proliferation, migration, and invasion [19, 20]. Moreover, researchers have found EpCAM-positive tumor cells in peritoneal effusions from multiple types of epithelial cancers [13]. Therefore, EpCAM is a promising target for treating MA [15–18].

Studies have shown that Bispecific antibodies (BsAbs)can act in either a combinatorial or an obligate manner, with the latter meaning that the same mechanism of action cannot be achieved by simply combining antibodies [21–24]. CD3 is a transmembrane protein complex primarily expressed on the surface of T lymphocytes, playing a critical role in T cell activation by transmitting signals from the T cell receptor (TCR) upon antigen recognition. Over the past two decades, T-cell engagers (TCEs) that specifically bind to a tumor surface antigen and the CD3ε chain of the T-cell receptor have dominated this class of bispecific antibodies; several hundred TCEs have been described, with more than 100 advancing to clinical development [25–28]. A major challenge in the clinical development of TCEs involves the occurrence of cytokine release syndrome, which is largely caused by the activation of on-target T-cells [29, 30]. Although this can often be managed using pretreatment using steroids and by step-up dosing, recent efforts have focused on developing CD3ε antibodies with a decrease in CD3ε affinity to uncouple T-cell killing from cytokine secretion [31–37].

Catumaxomab is a bispecific chimeric monoclonal antibody derived from mouse and rat sources that targets EpCAM and human cluster of differentiation 3 (CD3). It was approved by the EMA in 2009 for treating MA because of its favorable efficacy in reducing the frequency of puncture or drainage [38]. However, due to commercial reasons, it was withdrawn from the European market in 2017 and reapproved by the European Commission in 2025 (https://www.ema.europa.eu/). This EpCAM×CD3 bispecific antibody facilitates the binding of immune cells to tumor cells, boosting the targeted immune response and increasing immune cytotoxicity against tumor cells. Moreover, catumaxomab binds to CD3 on T cells, triggering their activation and proliferation, causing tumor-killing molecules such as tumor necrosis factor-alpha (TNF-α), interferon-gamma (IFN-γ), perforin, and granzyme B to be released [38, 39]. Additionally, catumaxomab exhibits antibody-dependent cell-mediated cytotoxicity and complement-dependent cytotoxicity against tumor cells [38, 39].

The novel human-mouse chimeric monoclonal antibody M701 targets the same antigens as catumaxomab but features a restructured design and robust chemical, manufacturing, and controls(CMC) properties via the CHO cell expression system. With optimized EpCAM and CD3 affinities, M701 is designed for treating MA and malignant pleural effusions caused by solid epithelial tumors. This bispecific antibody has shown significant inhibitory effects on tumor cells derived from MA in preclinical studies, indicating it is a targeted therapeutic approach [40]. A Phase I clinical study was performed on patients with epithelial tumor and MA, and a Phase Ib study was conducted on lung cancer patients with malignant pleural effusion. Both studies showed a favorable safety profile and promising therapeutic efficacy in controlling MA and malignant pleural effusion [41, 42]. Recently, a Phase II clinical trial was conducted to further evaluate the safety and efficacy of IP administration of M701 in patients with advanced epithelial solid tumors complicated by MA.

## Methods

### Anti-EpCAM and anti-CD3 BsAb design

Anti-EpCAM and anti- CD3 BsAb (M701) is a Fab-scFv-Fc bispecific antibody (BsAb), the Fab moiety is anti-EpCAM, and the scFv moiety is anti-CD3. The anti-EpCAM monovalent unit and the anti-CD3 single-chain unit of M701 were from the anti-EpCAM variable regions of AMG110 (Amgen) and L2K, respectively. The mutations in the CH3 domains of the human IgG1 Fc fragment included T366W-Y407A (knobs-into-holes pair), L368R-K409D (ionic bond “salt bridge”), and D399K-K392D (second salt bridge). M701 was stably expressed in the CHO-S (Gibco) expression system. The purification process was similar to that used to purify classical monoclonal antibodies, including depth filtration, affinity chromatography, low pH chromatography, ion-exchange chromatography, nanofiltration, and ultrafiltration/diafiltration (UF/DF).

### Molecular weight determination by LC-MS

Reverse-phase High-Performance Liquid Chromatography (HPLC) was performed using an ultra-high-performance (UPLC) system (Vanquish, Thermo), coupled to an orbitrap mass spectrometer (Q Exactive Plus, Thermo).

### Cell binding assay

The HCT116 cells were incubated with serially diluted M701 or with anti-EpCAM monoclonal antibody for 1 h at room temperature and then incubated with PE-conjugated anti-human IgG Fc (secondary antibodies) for 30 min in the dark. The binding activity was evaluated by flow cytometric analysis. The same method was used to measure the affinity of M701 and L2K (a reference antibody) for human T cells. The dissociation constant( Kd )was calculated using the software GraphPad Prism. To measure the M701-induced cell bridging, EpCAM-positive NCI-N87 cells were labeled with 2.5 µM CFSE (Invitrogen), and CD3-positive cytokine-induced killer cells (CIKs) were labeled with 0.2 µM PKH26 (Sigma). The cells were washed three times and mixed with each other at a ratio of 1:1. The mixtures were incubated with serially diluted antibodies (0 to 10 µg/mL) for 30 min at 37 °C in a 96-well round bottom plate (Corning). M701-mediated cell bridging was evaluated by conducting flow cytometry analysis and is presented as the percentage of cells in the upper right quadrant of an FL1 vs. FL2 scatter plot, which represents the CFSE-PKH26-double-positive population.

### In vitro cytotoxicity assay

CIKs were utilized as effector cells to evaluate the in vitro efficacy of M701. Peripheral blood mononuclear cells (PBMCs) from healthy donors were isolated using Ficoll-Hypaque (Sigma) density gradient centrifugation, according to the manufacturer’s protocol. The isolated PBMCs were then co-cultured with anti-CD3 monoclonal antibody, IFN-γ, IL-2, and IL-1α for 14 days to generate CIKs. The bioactivity of M701 was assessed using a fluorescence-activated cell sorting (FACS)-based cytotoxicity assay. Target cells included EpCAM-high cancer cell lines (HCT116, colorectal carcinoma; OVCAR-3, ovarian adenocarcinoma; KATO-III, gastric carcinoma), EpCAM-low cancer cell lines (SK-OV-3, ovarian adenocarcinoma; MDA-MB-231, breast adenocarcinoma), and the EpCAM-negative cancer cell line U87 (glioblastoma). In the assay, 2.0 × 10⁴ CFSE-labeled target cells were co-cultured with CIK effector cells at an effector-to-target (E: T) ratio of 5:1 in 96-well flat-bottom plates (Corning) with serial dilutions of M701 or control antibodies for 24 h at 37 °C in a 5% CO₂ incubator. After incubation, cells were harvested and stained with propidium iodide (PI, Sigma) to assess apoptotic/necrotic populations by flow cytometry.

### Animal studies

Mouse Strains and Housing: Female NOD/SCID and C57BL/6 mice (7–8 weeks old) were purchased from Beijing HFK Bioscience Co., Ltd. Xenograft subcutaneous tumor model: A total of 1.0 × 10⁷ HCT116 cells and 1.0 × 10⁷ CIK cells were mixed and inoculated subcutaneously into the right dorsal flank of 30 NOD/SCID mice. Within 2 h of inoculation, the mice were randomly assigned to three experimental groups and three control groups (*n* = 5 per group). Mice in experimental groups received intravenous bolus injections of M701 (0.1, 0.5, or 2.5 mg/kg) into the lateral tail vein. Mice in the control groups were treated with anti-EpCAM monoclonal antibody (mAb) (2.5 mg/kg), anti-CD3 isotype (2.5 mg/kg), or saline. Treatments were repeated on days 2 and 4. Tumor volumes were measured every three days using a digital caliper, and tumor volume (mm³) was calculated using the formula: ½ × (length × width²).

Syngeneic Ascites Tumor Model: The CT26-mEpCAM cell line, a CT26-derived cell line transfected to express high levels of murine EpCAM, was used in this model. M708, a surrogate of M701, binds both murine EpCAM and murine CD3. The CT26-mEpCAM murine model was established by subcutaneously inoculating 3.0 × 10⁶ CT26-mEpCAM cells into the right dorsal flank of C57BL/6 mice. Starting on day 0 and continuing on days 2, 4, 7, 9, and 11, mice received IP bolus injections of 0.025 mg/kg M708, 0.25 mg/kg M708, 2.5 mg/kg M708, 0.25 mg/kg anti-mEpCAM mAb, 0.25 mg/kg anti-mCD3 isotype, a combination of 0.25 mg/kg anti-EpCAM and 0.25 mg/kg anti-mCD3 isotype, or vehicle (*n* = 10 per group). Mice were euthanized when tumor volume reached 2000 mm³ or at the conclusion of the study.

### Phase II trial design

We conducted an open-label, multicenter, randomized, and controlled phase II trial, M70102, in 34 hospitals in China between December 2022 and July 2024 (ClinicalTrials.gov identifier: NCT06266091). The study was conducted following the guidelines of the Declaration of Helsinki and approved by the institutional review board of all involved hospitals. All patients signed informed consent before participating in any study-related procedure.

### Trial population

Patients aged 18–75 years diagnosed with gastrointestinal or ovarian cancers with moderate-to-large-volume symptomatic MA were enrolled. Gastric and colorectal cancer patients had failed at least two lines of systemic treatment, while ovarian cancer patients were platinum-resistant. Additionally, only patients with an Eastern Cooperative Oncology Group (ECOG) performance status of 0–2, life expectancy ≥ 8 weeks and adequate bone marrow function, defined as absolute neutrophil count ≥ 1.5 × 10^9^/L, platelet count ≥ 80 × 10^9^/L, hemoglobin ≥ 9.5 g/dL, serum albumin ≥ 28 g/L, and relative lymphocyte count (RLC) ≥ 10% in peripheral blood, were allowed to participate. Patients whose serum creatinine level was > 1.5 times the upper limit of normal were excluded. A two-week washout period before cycle 1, day 1 was mandatory if the patients were administered with any IP chemotherapy.

### Trial intervention

The patients were randomly assigned to two groups at a ratio of 1:1. In the M701 group, patients received paracentesis and IP infusions of 50, 400, 400, and 400 µg of M701 on days 1, 4, 11, and 18, respectively. Additional M701 IP infusions could be administered every two weeks without requiring punctures or drainage until the MA was intolerant or patients died. In the control group, patients received paracentesis alone as needed from days 1 to 18. Both arms received systemic tumor treatment as determined by investigators following the guidelines. After day 18, no puncture or drainage was allowed for the patients in either arm until the MA was intolerant. The patients in the control group were allowed to transfer to the M701 group and receive the M701 IP infusion after they reached the primary endpoint.

### Endpoints and statistical analysis

The primary endpoint was PuFS, defined as the time to the first re-puncture or drainage due to intolerance of MA or death after the last drainage from days 1 to 18, whichever occurred first. The criteria for ascites intolerance include: (1) The patient complains that he/she cannot tolerate the ascites; (2) A large amount of ascites is confirmed by B-ultrasound, with the maximum depth of ascites ≥ 4.5 cm; (3) The investigator determines that the patient’s total score for ascites symptoms and signs exceeds 7 points ( Likert 4-point scale, see Appendix 1). To define the intolerance of ascites, Criterion 1 must be met. For Criterion 2 and Criterion 3, meeting either one is sufficient. The secondary efficacy endpoints included OS, progression-free survival (PFS) for the target lesion according to the RECIST 1.1 criteria, and the best objective response rate (ORR) of MA following the WHO criteria.

Safety was assessed based on adverse events (AEs) reported during the study. All AEs were coded using Medical Dictionary for Regulatory Activities (MedDRA 27.0) and graded according to the Common Terminology Criteria for Adverse Events (CTCAE) V5.0. Cytokine release syndrome (CRS) was the only adverse event of special interest (AESI) listed in this study.

The sample size was determined by conducting log-rank tests. The assumption was that the median PuFS of the control group was 18 days, and the hazard ratio (HR) between the experimental group and control group was 0.5. Assuming a power of 80%, alpha = 5%, and a 10% dropout rate, a sample size of 60–80 patients were required.

Fisher’s exact test was used to analyze nominal variables, and the Mann-Whitney U test for continuous variables. Survival curves were estimated using the Kaplan-Meier method. All data were analyzed using Statistical Analysis System software (SAS^®^ 9.4 or higher version).

Statistical analysis was performed using Prism 6 (GraphPad Software Inc., La Jolla, CA) for both cell and animal studies The differences between groups were determined by conducting two-sided Student’s t-tests. All data were presented as the mean ± SD. All results were considered to be statistically significant at *P* < 0.05.

### Pharmacokinetics and immunogenicity

Blood and ascites samples were collected to evaluate the pharmacokinetics and immunogenicity of M701. The pharmacokinetic analysis set (PKAS) and immunogenicity analysis set (IS) included all patients who received at least one dose of M701 infusion and from whom adequate drug concentration or anti-drug antibody (ADA) measurements were obtainable during the trial. Blood samples for pharmacokinetic and immunogenicity analysis were collected on days 1, 4, 18, 46, and 88 and every two treatment cycles 1 h before M701 infusion. Ascites samples for pharmacokinetic and immunogenicity analysis were collected on days 1, 18, 32, 46, and 88 and every two treatment cycles 24 h before M701 infusion. The serum and ascites concentrations of M701 or ADA were measured using validated methods.

## Results

### Characteristics of M701

**Fig. 1:**
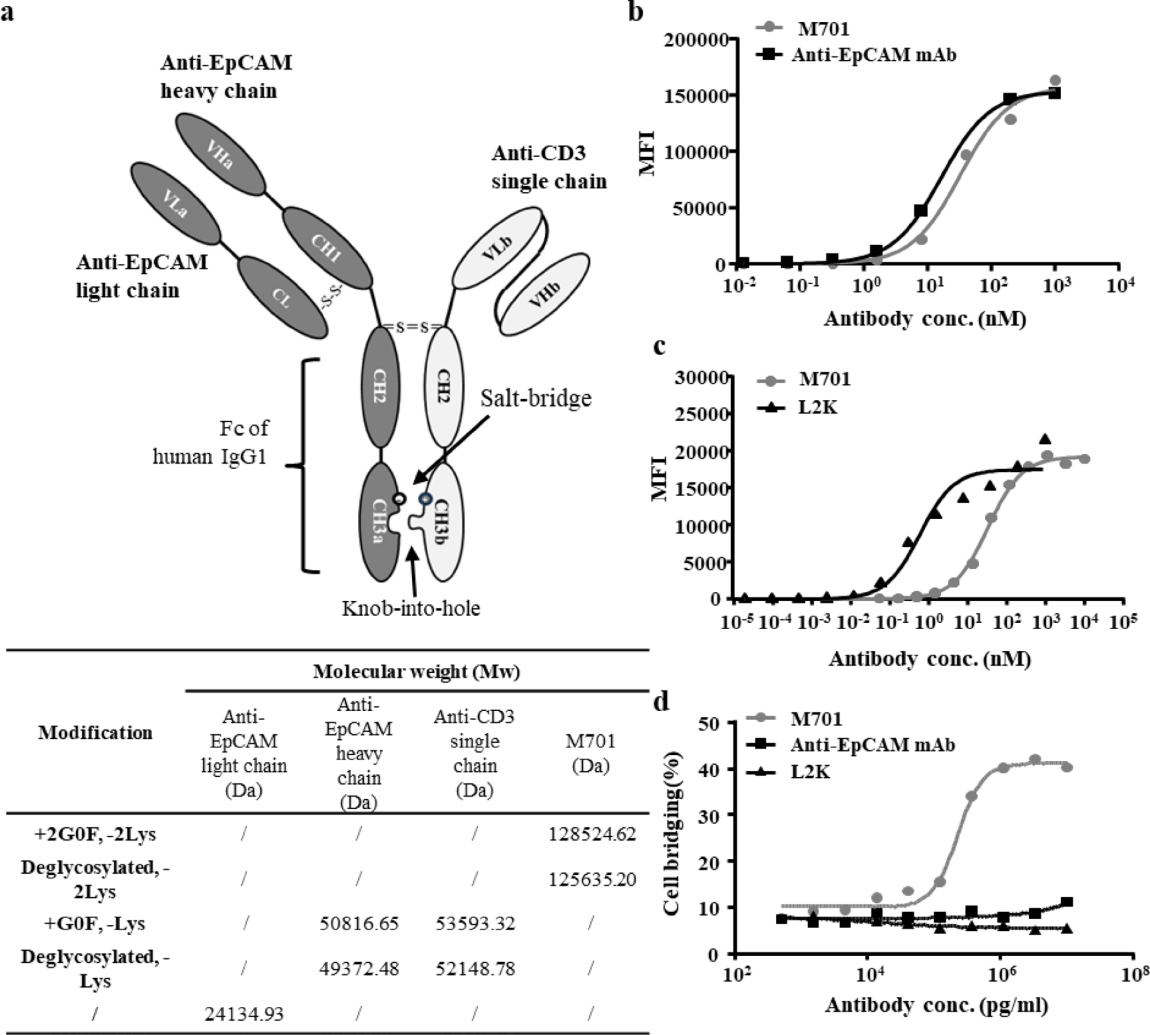
Characteristics of M701. **a** A schematic diagram and molecular weight (Mw) of M701. M701 consists of a monovalent unit and a single-chain unit. The anti-EpCAM monovalent unit consists of an anti-EpCAM heavy chain and an anti-EpCAM light chain conjugated by a disulfide bond. The anti-CD3 single-chain unit had a VHb-linker-VLb-Fc structure. Two disulfide bonds were formed between the monovalent unit and the single-chain unit. VHa/VLa denotes the variable regions that belong to the monovalent unit, whereas VHb/VLb refers to the variable regions associated with the single-chain unit, and CH3a/CH3b refers to the distinct modifications of the CH3 domain that are introduced to increase heterodimer formation. The modifications include knob-into-hole and salt-bridge. The glycosylated and deglycosylated Mw of each unit of M701 was determined by LC-MS (liquid chromatography-mass spectrometry). G0F is the predominant glycoform inr recombinant human IgG1 antibodies produced in CHO cells. Lys is C-terminal lysine residues at the Fc region of antibodies which is incompletely removed by carboxypeptidase B during antibody production. **b** The affinity of M701 for the anti-EpCAM moiety and the parent anti-EpCAM mAb for HCT116 cells; M701 Kd = 32 nM, and the anti-EpCAM mAb Kd = 16 nM. **c** The affinity of M701 for the anti-CD3 moiety and the parent antibody L2K for human T cells; M701 Kd = 33.7 nM, and L2K Kd = 0.6 nM.** d** 701-mediated cell bridging. The cell bridging of NCI-N87 cells (stained with CFSE) with Cytokine-induced killer cells (CIKs) cells (stained with PKH26) without or with M701 are shown

M701 consists of two distinct units, including a monovalent unit comprising a heavy chain/light chain pair and a single-chain unit (Fig. 1a). In the single-chain unit, the single-chain variable fragment (scFv) is constructed in a variable heavy chain (VH)-variable light chain (VL) orientation, with VH and VL connected in-frame by a (Gly_4_Ser)_3_ linker, and the scFv is fused to the human IgG1 Fc fragment to form the single-chain This design prevents mismatched pairing and improves the manufacturability of the antibody.

The glycosylated and deglycosylated intact masses of M701 were 128.5 kDa and 125.6 kDa, respectively. Under reducing conditions, the molecular weight (Mw) of the light chain was 24.1 kDa, the glycosylated Mw of the heavy chain was about 50.8 kDa and the single-chain was 53.6 kDa, the deglycosylated Mw of the heavy chain was about 49.4 kDa, and the single-chain Mw was 52.1 kDa (Fig. 1a).

The mutations in the Fc fragments of M701 were based on a salt bridge and knobs-into-holes (KIHs). The specific modifications were T366W, K392D, and K409D on the Fc of the monovalent unit and L368R, D399K, and Y407A on the Fc of the single-chain unit.

### M701 exhibited moderate affinity and dose-dependent cell bridging

The cell binding activity of M701 was evaluated by conducting flow cytometry assays. The results indicated that the affinity of M701 (Kd = 32 nM) was approximately half that of the anti-EpCAM mAb (Kd = 16 nM) for HCT116 cells (Fig. 1b) and significantly weaker than that of L2K for human T cells (M701 Kd = 33.7 nM, L2K Kd = 0.6 nM) (Fig. 1c). Additionally, M701-mediated recruitment of CD3-positive cells to EpCAM-positive cells was investigated. Cytokine-induced killer (CIK)cells expressing CD3 were labeled with PHK26, and EpCAM-positive NCI-N87 cells were labeled with CFSE. The proportion of double-positive cells was less than 10% in the absence of M701 treatment (Fig. 1d). In contrast, in the presence of 10 µg/mL M701, up to 40% of the total cell population was double-positive for M701-mediated cell bridging. The enhanced level of cell bridging mediated by M701 was also dose dependent. Neither the anti-EpCAM mAb nor L2K could mediate the cell bridging at any concentration tested.

### M701 potently redirected Lysis to EpCAM-positive tumor cells

**Fig. 2:**
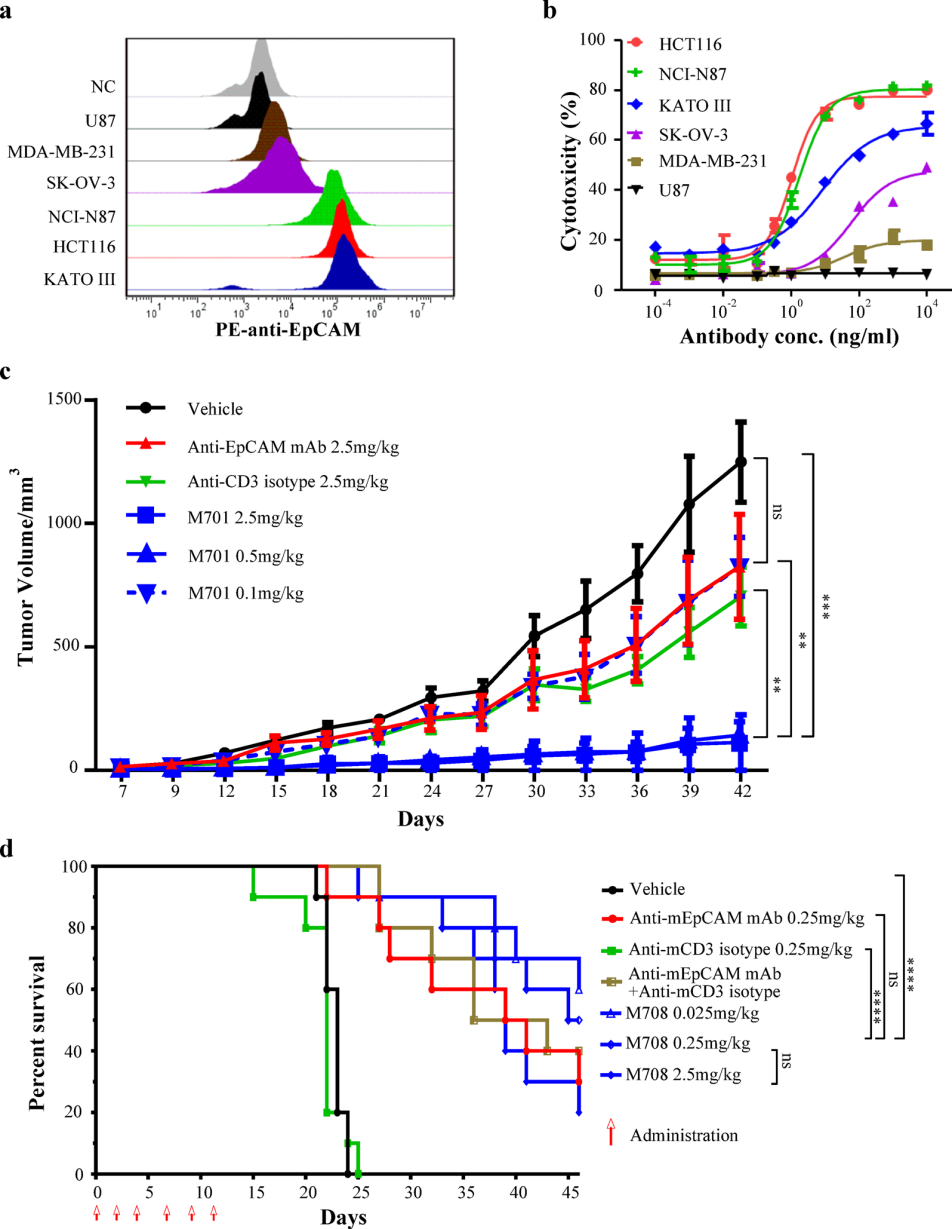
In vitro cytotoxicity and in vivo antitumor efficacy of M701. **a** Detection of EpCAM expression in different tumor cell lines via fluorescence-activated cell sorting (FACS). NC was a negative control by using a lymphoma cell line Raji. **b** M701-mediated redirected lysis of cytokine-induced killer cells (CIKs) to six cancer cell lines with different EpCAM expression levels. CIKs were effectors (E) and tumor cells were targets (T). All cell lines were incubated for 24 h in the presence of different concentrations of M701 with CIKs at an E: T ratio of 5:1. M701 inhibited the proliferation of tumor cells HCT116, NCI-N87, KATOIII, SK-OV-3 and MDA-MB-231 with EC50 values of 1.1, 1.8, 8.5, 58.7 and 37.8 ng/mL, respectively. **c** HCT116 xenograft model in CIK-reconstituted NOD/SCID mice treated with M701 (i.v., administered on days 0/2/4). Tumor volumes (day 42, mm³, mean ± SD, *n* = 5): vehicle (1249.89 ± 364.88), anti-EpCAM mAb (827.54 ± 475.95), anti-CD3 isotype (704.03 ± 269.78), M701 2.5 mg/kg (112.54 ± 251.64), 0.5 mg/kg (145.15 ± 124.84), 0.1 mg/kg (828.17 ± 267.18). **d** CT26-mEpCAM ascites model in C57BL/6 mice treated with M708 (i.p., administered on days 0/2/4/7/9/11). M708 is a murine surrogate bsAb (anti-murine EpCAM and murine CD3) of M701. Survival rates (day 46): vehicle (0%), anti-mCD3 isotype 0.25 mg/kg (0%), anti-mEpCAM mAb 0.25 mg/kg (30%), the combination of anti-mEpCAM mAb 0.25 mg/kg and anti-mCD3 isotype 0.25 mg/kg (40%), M708 2.5 mg/kg (20%), 0.25 mg/kg (50%), 0.025 mg/kg (60%). Statistics: multiple t-test (**c**); Log-rank test (**d**). * *p* < 0.05, ** *p* < 0.01, *** *p* < 0.001, **** *p* < 0.0001, ns (not significant). (Abbreviations: bsAb, bispecific antibody; CIK, cytokine-induced killer cell; FACS, fluorescence-activated cell sorting; i.p., intraperitoneal; i.v., intravenous; mAb, monoclonal antibody)

We evaluated the cytotoxic effects of M701, mediated by CIKs, on cancer cells expressing different levels of EpCAM. Target cells with different EpCAM expression levels (Fig. 2a) were incubated with increasing concentrations of M701 and CIKs at an E: T ratio of 5:1. M701 showed potent cytotoxicity against HCT116, NCI-N87, and KATO III cells (Fig. 2b), all of which express high levels of EpCAM. However, M701 displayed minimal cytotoxic effects on SK-OV-3 and MDA-MB-231 cells, which express very low levels of EpCAM (Fig. 2b). Additionally, M701 did not induce cytotoxicity in EpCAM-negative U87 cells, confirming the specificity of the M701-mediated cell lysis (Fig. 2b).

M701 specifically induces potent cytotoxicity in EpCAM-positive tumor cells, with minimal effects on EpCAM-negative cells, highlighting its potential as a targeted therapeutic agent for EpCAM-expressing cancers.

### M701 and surrogate M708 exhibited potent antitumor efficacy in solid and Ascites tumor models

In a xenograft subcutaneous tumor model, M701 demonstrated superior efficacy in inhibiting the growth of human colon cancer cells (HCT116) in NOD-SCID mice compared to control antibodies (Fig. 2c).

As a surrogate of M701, M708 possesses the same Fab-scFv-Fc structure and binds both murine EpCAM and CD3. In a syngeneic ascites tumor model, IP injection of M708 significantly prolonged the survival of mice with malignant ascites in a dose-dependent manner at low, medium, and high doses (Fig. 2d).

This indicates that a BsAb targeting EpCAM and CD3 has a strong inhibitory effect on EpCAM-positive tumors and the malignant ascites caused by these tumors.

### Baseline characteristics of patients in the phase II trial

**Fig. 3:**
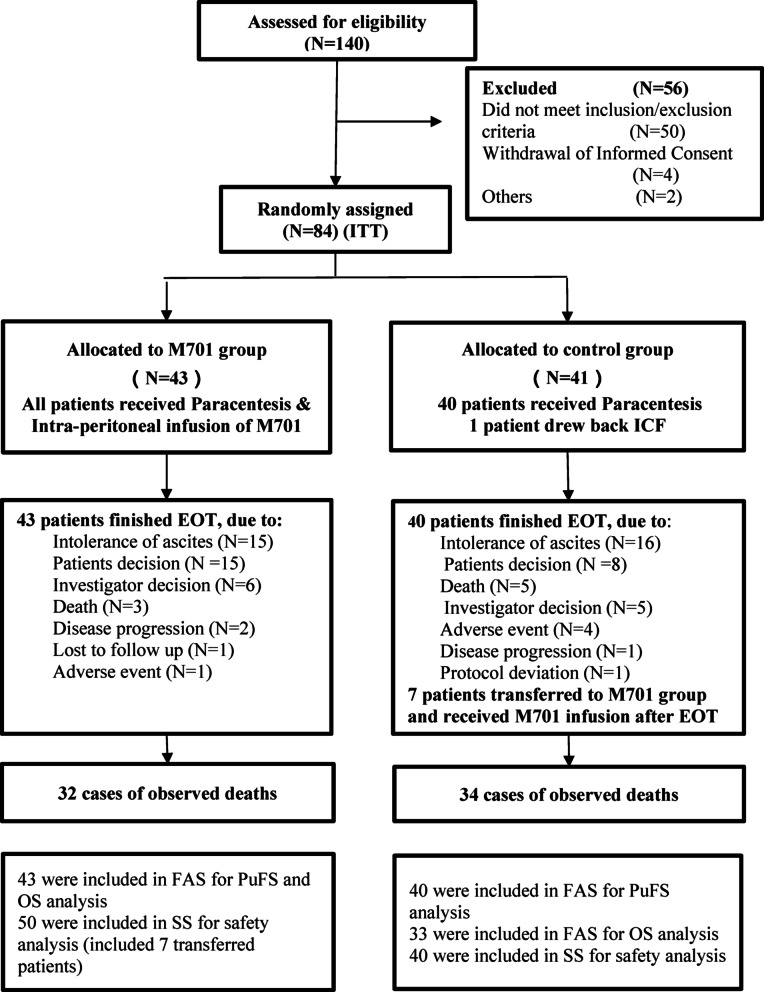
CONSORT flow diagram (Only results for the intent-to-treat and safety populations are included in this article. 7 patients in control group transferred to the M701 group after they were intolerant of the ascites.)

From November 2021 to July 2024, 84 patients were enrolled, 43 patients were assigned to the M701 group, 41 patients were assigned to the control group, and one patient in the control group withdrew from the study after initial screening. In total, 83 patients who were administered at least one M701 infusion in the M701 group or one therapeutic paracentesis in the control group were included in the full analysis set (FAS). According to the protocol, seven patients in the control group were transferred to the M701 group to receive M701 via IP infusion. The CONSORT flow diagram is shown in Fig. 3. The baseline characteristics of the intent-to-treat (ITT) population are shown in Table 1, and the patients and disease characteristics were balanced between the M701 group and the control group. Most patients in both arms had gastric cancer, accounting for 48.8% of those in the M701 group and 45.0% of those in the control group. The percentage of patients with prior paracentesis was 65.1% in the M701 group and 65.0% in the control group, and the median number of patients with prior paracentesis was one and two in the M701 and control groups, respectively. The median number of prior systemic treatment lines was two in both groups. A total of 30.2% and 32.5% of patients in the M701 and control groups, respectively, received more than three lines of systemic treatment. The systemic therapies were well balanced between those two arms. The combined systemic anticancer therapy during the study period were listed in Table 2.

**Table 1:** Patient characteristics in ITT (*N* = 84)

Characteristic		ITT Set	
		M701 (*N* = 43)	Control (*N* = 41)
Age(years)	Mean (SD)	54.8 (8.99)	54.4 (10.64)
	Median (P25,P75)	54 (50.0–59.0)	54 (49.0-62.5)
	≥ 60	10 (23%)	14 (34%)
Gender	Male	14 (33%)	14 (34%)
	Female	29 (67%)	27 (66%)
ECOG	0	4 (9%)	2 (5%)
	1	34 (80%)	34 (83%)
	2	4 (9%)	5 (12%)
Cancer Type	Gastric	21 (49%)	19 (46%)
	Ovarian	13 (30%)	13 (32%)
	Colorectal	8 (19%)	8 (20%)
	Fallopian tube cancer	1 (2%)	0
	Primary peritoneal cancer,	0	1 (2%)
If maintain the system treatment regimen after enrolled	Yes	3 (7%)	3 (7%)
	No	40 (93%)	38 (93%)
Clinical Stages	II	1 (2%)	1 (2%)
	III	10 (23%)	3 (7%)
	IV	30 (70%)	37 (90%)
	Unknown	2 (5%)	0
Previous treatment	IP treatments	24 (56%)	21 (51%)
	Paracentesis	27 (63%)	25 (61%)
Previous paracentesis frequency (%)	0	37.2%	46.3%
	1–3	51.2%	31.7%
	≥ 4 times	11.6%	17.1%
	Unknown	0	4.9%

**Table 2:** Combined systemic anticancer therapy during the study period

	M701 *N* = 43	Control *N* = 40	Total *N* = 83
Systemic anticancer therapy	41 (95.3%)	40 (100%)	81 (97.6%)
Apatinib Mesylate	8 (18.6%)	5 (12.5%)	13 (15.7%)
Nivolumab	13 (30.2%)	13 (32.5%)	26 (31.3%)
Regorafenib	5 (11.6%)	4 (10.0%)	9 (10.8%)
Fruquintinib	2 (4.7%)	4 (10.0%)	6 (7.2%)
Paclitaxel Albumin-Bound	2 (4.7%)	3 (7.5%)	5 (6.0%)
Doxorubicin Hydrochloride Liposome	11 (25.6%)	11 (27.5%)	22 (26.5%)
Others	9 (20.9%)	0	9 (10.8%)

In accordance with Chinese clinical practice guidelines, Apatinib Mesylate and Nivolumab were recommended for patients with gastric cancer; Regorafenib and Fruquintinib for patients with colorectal cancer; and Paclitaxel Albumin-Bound and Doxorubicin Hydrochloride Liposome for patients with platinum-resistant ovarian cancer

For patients in the study who experienced systemic tumor progression but still tolerate MA, other systemic therapeutic agents were allowed to switched to. In the M701 group, 9 subjects were evaluated as having systemic tumor progression during the study and then switched to new systemic therapeutic agents, including monotherapy regimens such as Longsurf, Capecitabine, Irinotecan Hydrochloride, Gemcitabine, Docetaxel, or Bevacizumab. Subjects in the control group usually could no longer tolerate MA before the occurrence of systemic tumor progression

### Treatment outcomes

**Fig. 4:**
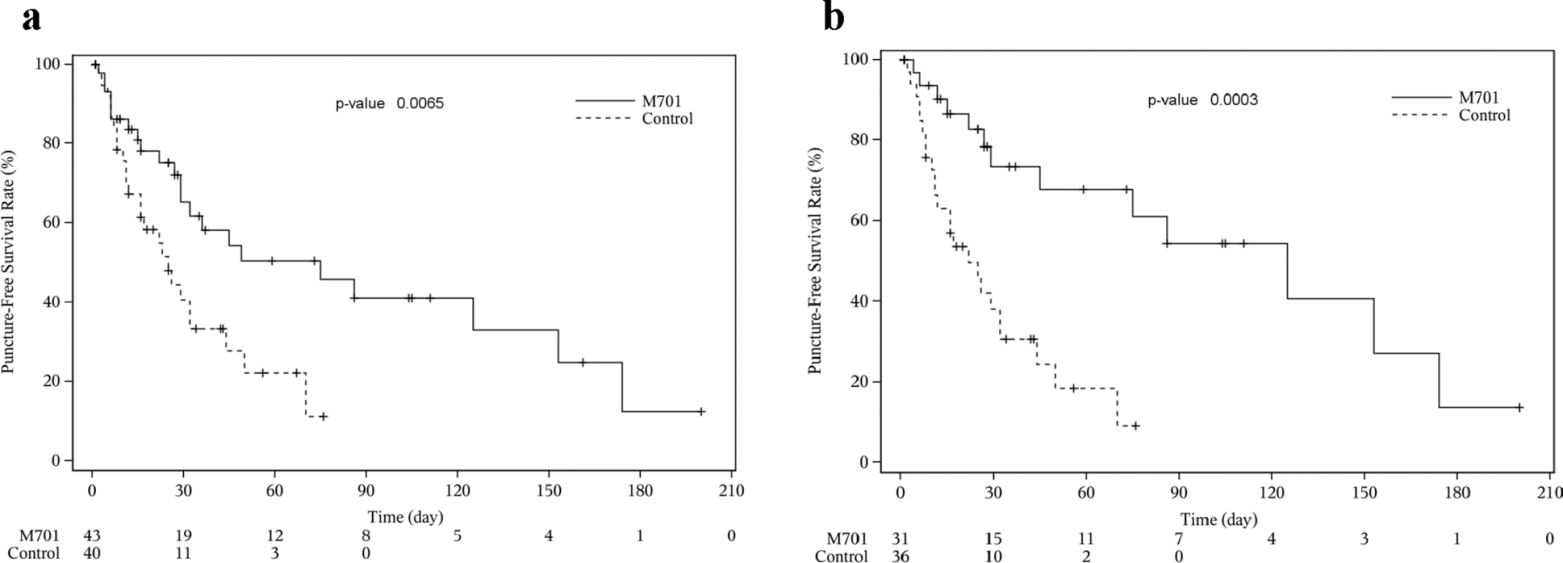
Kaplan-Meier estimates of puncture-free survival. **a** Puncture-free survival in the FAS population. **b** Puncture-free survival in the RLC ≥ 13% population

To evaluate the efficacy of M701 in controlling MA, PuFS was set as the primary endpoint and directly reflected the ability of treatment to prevent the re-accumulation of ascites in the peritoneal cavity. Median PuFS was significantly longer in the M701 group than in the control group in the FAS population (75 vs. 25 days) (Fig. 4a), with an HR of 0.43 (95% CI: 0.22, 0.81) and *p* = 0.0065. The one-month PuFS rates were 65.2% in the M701 group and 40.6% in the control group, whereas the two-month PuFS rates were 50.3% in the M701 group and only 22.1% in the control group (Table 3). A significant difference in the two-month PuFS rates was found between the two groups (*p* = 0.001), indicating that compared to the control group, the M701 group had a significantly longer time without the need for peritoneal drainage.

**Table 3:** PuFS in the FAS and RLC ≥ 13% of populations

N	FAS population		RLC ≥ 13% population	
	M701	Control	M701	Control
	43	40	31	36
Median PuFS (95%CI), Day^a^	75 (29–153)	25 (12–32)	125 (45–174)	22 (11–32)
*P* value (Log-rank test)^b^	0.0065		0.0003	
Hazard Ratio(95%CI)^c^	0.43 (0.22, 0.81)		0.23(0.10, 0.55)	
Half a month PuFS rate (95%CI)^a^	80.8 (65.2, 89.9)	67.2 (49.5, 79.9)	86.6 (68.1, 94.8)	63.1 (44.3, 77.1)
1-month PuFS rate (95%CI)^a^	65.2 (47.1, 78.4)	40.6 (23.6, 56.9)	73.4 (51.5, 86.6)	38.2 (21.1, 55.2)
2-month PuFS rate (95%CI)^a^	50.3 (31.8, 66.3)	22.1 (8.2, 40.2)	67.8 (44.7, 82.9)	18.3 (5.5, 37.0)

a. Based on the Kaplan-Meier estimates

b. Based on the Log-rank test

c.Based on the Cox regression

**Fig. 5:**
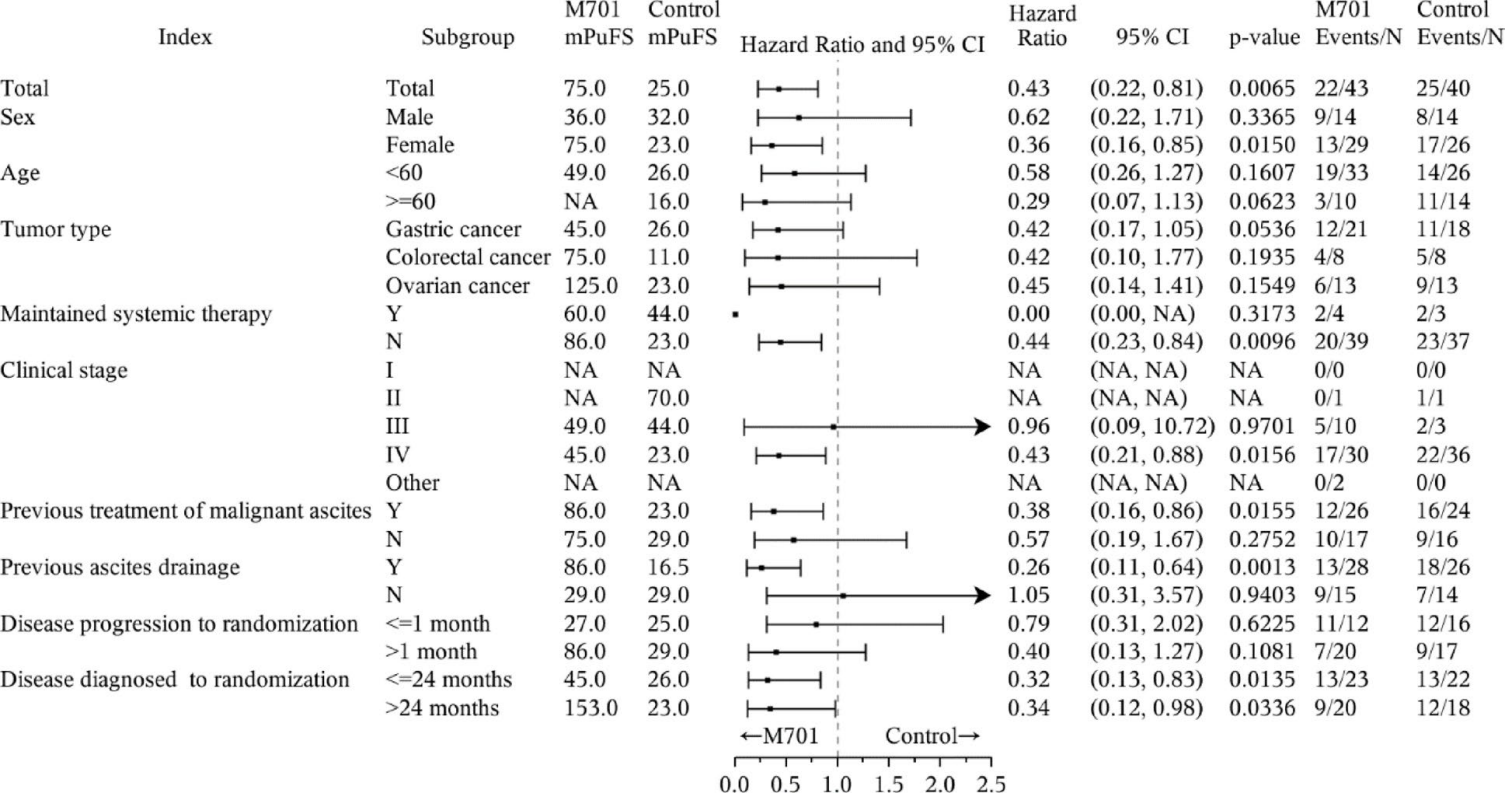
Puncture-free survival forest plot of the FAS population. Definition of previous treatment of malignant ascites: With a record of intraperitoneal anti-tumor drug perfusion in the patient’s past medical history. Definition of previous ascites drainage: With a record of abdominal paracentesis and drainage in the patient’s past medical history

The PuFS in different subpopulations was also analyzed. The different subpopulations benefited from M701 treatment, regardless of sex, age, type of cancer, clinical stage, previous treatment history, and time point of disease diagnosis. Compared to the women in the control group, women with Stage IV disease who previously received local chemotherapy or paracentesis significantly benefited from M701 treatment (Fig. 5).

An in vitro study revealed that M701 could bridge T lymphocytes to EpCAM-positive tumor cells, which activated T lymphocytes to kill tumor cells. Therefore, the rationale that patients with high T lymphocyte counts respond better to M701 treatment was verified in the subpopulation with a high RLC in whole blood cells. In the population with RLC ≥ 13% at baseline, the M701-treated patients showed an even greater PuFS benefit than individuals in the control group (Fig. 4b). The median PuFS were 125 days and 22 days, respectively (*p* = 0.0003, HR = 0.23 (0.10, 0.55)) (Table 3).

**Fig. 6:**
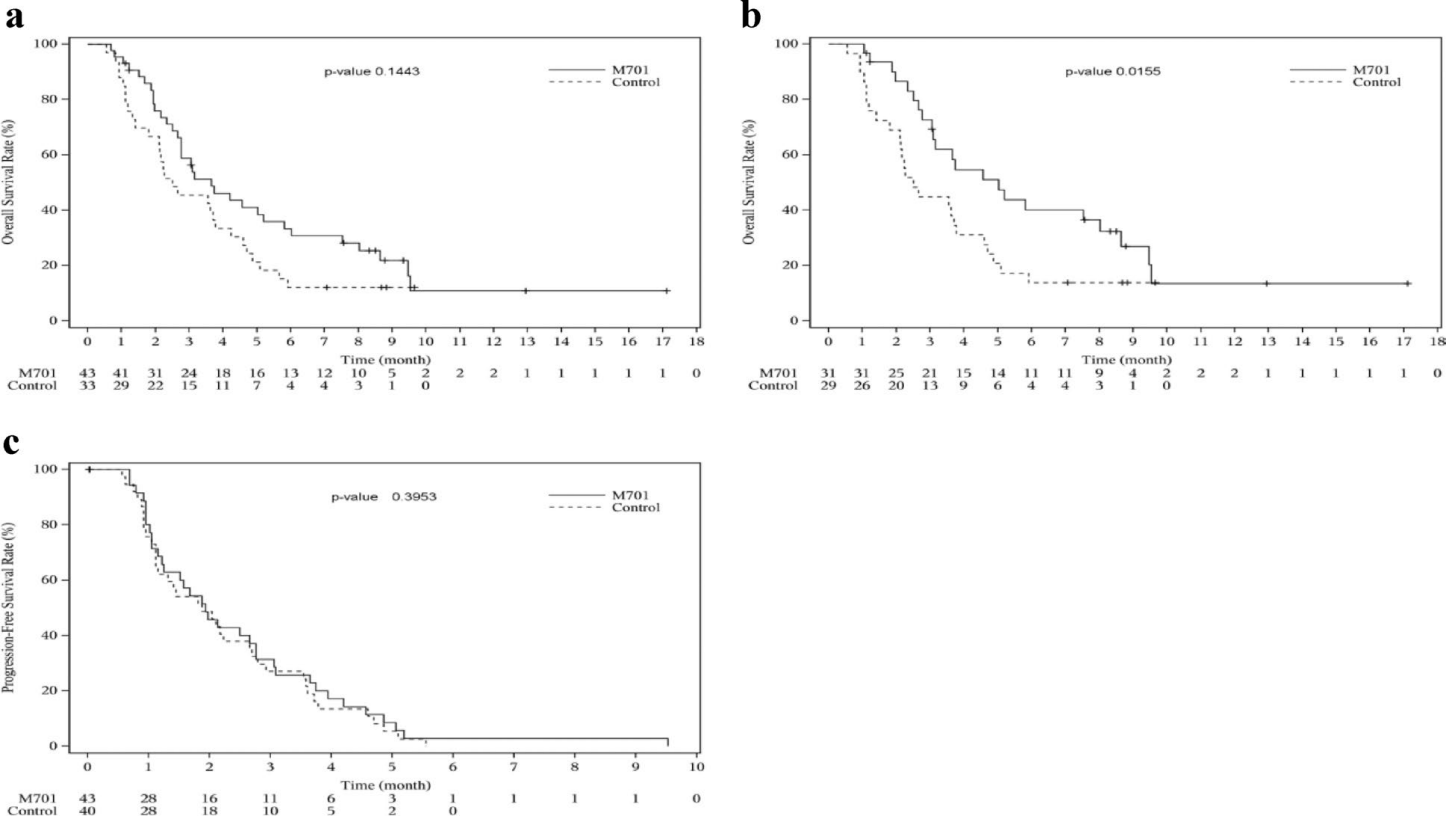
Kaplan-Meier estimates of Progression-free Survival and Overall survival.** a** Overall survival in the FAS population;** b** Overall survival in the RLC ≥ 13% subpopulation; (c) Progression-free survival in the FAS population

Tumor response to systemic therapy was not found in either group (Objective response rate = 0%). The PFS in those two groups was not significantly different (HR = 0.80 and *p* = 0.40) (Fig. 6c).

The final survival analysis suggested that treatment with M701 provided a trend toward survival benefit with HR = 0.68 and *p* = 0.1443 (Fig. 6a). The six-month survival rate in the M701 group was considerably higher than that in the control group (33.3% vs. 12.1%) (Table 4). In the population with RLC ≥ 13% at baseline, the M701-treated patients received significant benefits in overall survival (HR = 0.46 and *p* = 0.0155) (Fig. 6b).

**Table 4:** Overall survival in the FAS and RLC ≥ 13% of populations

N	FAS population^d^		RLC ≥ 13% population^d^	
	M701	Control	M701	Control
	43	33	31	29
Median OS(95%CI), Daya	110 (82, 177)	76 (55, 116)	152 (94, 244)	76 (55, 116)
P value(Log-rank test)b	0.1443		0.0155	
Strata Hazard Ratio (95%CI)c	0.68 (0.40, 1.15)		0.46 (0.24, 0.88)	
2-month survival rate(95%CI)a	75.9 (59.9, 86.3)	66.7 (47.9, 80.0)	86.5 (67.9, 94.7)	69.0 (48.8, 82.5)
3-month survival rate(95%CI)a	58.8 (42.3, 72.0)	45.5 (28.2, 61.2)	72.7 (52.7, 85.3)	44.8 (26.5, 61.6)
6-month survival rate(95%CI)a	33.3 (19.4, 47.8)	12.1 (3.8, 25.5)	40.1 (22.3, 57.3)	13.8 (4.3, 28.6)

a. Based on the Kaplan-Meier estimates

b. Based on the Log-rank test

c. Based on the Cox regression

d. The transferred patients in the control group were excluded

### M701 exposure and safety profiles

Among the patients in the M701 group and the control group transferred to the M701 group, 50 patients received between 1 and 16 infusions of M701 individually. The median number of infusions was four, with an average of five infusions per patient.

All patients in the control group received a puncture and an indwelling catheter. Among them, 85% required two or more drainages from days 1 to 18.

Although the IP infusion of M701 is a kind of local therapy, it is hypothesized that it will increase the risk to patients because of T lymphocyte activation. In this study, 50 subjects received at least one dose of M701 and systemic therapy, while 40 subjects in the control group received systemic therapy and paracentesis. Treatment emergent adverse events (TEAEs) occurred in 47 subjects (94.0%) in the experimental group and 37 subjects (92.5%) in the control group, with similar TEAE incidence rates between the two groups. The incidence of ≥ Grade 3 TEAE occurred in 52.0% and 55.0% of the subjects in the two groups, respectively. The results showed that 50% of subjects in both groups experienced serious adverse events (SAEs). The details of the safety profile are summarized in Table 5. Compared to paracentesis alone, the local infusion of M701 combined with systemic therapy did not increase the risk to patients.

**Table 5:** Overall adverse events in SS

	M701 (*N* = 50)	Control (*N* = 40)
	Case (incidence %)	Case (incidence %)
TEAE	47(94.0)	37(92.5)
M701 related AE	40 (80.0)	N/A
Systemic therapy related AE	36 (72.0)	30 (75.0)
≥ Grade 3 TEAE	26 (52.0)	22(55.0)
≥ Grade 3 M701 related AE	24 (48.0)	N/A
SAE	25(50.0)	20(50.0)
CRS	2 (4.0)	0 (0)

In the M701 group, the most common SAEs included gastrointestinal disorders (16.0%), laboratory examinations (14.0%), blood and lymphatic system disorders (12.0%), infections and infestations (10.0%), general disorders and administration site conditions (6.0%), and disorders related to metabolism and nutrition (6.0%). In the control group, the most common SAEs included gastrointestinal disorders (22.5%), infections and infestations (15.0%), disorders related to metabolism and nutrition (10.0%), and general disorders and administration site conditions (4.0%). The details of the SAEs are summarized in Table 6.

**Table 6:** List of patient SAEs in the safety set (≥ 5%)

By SOC	M701 group (*N* = 50)	Control group (*N* = 40)	Total (*N* = 90)
	Cases/Incidence(%)	Cases/Incidence(%)	Cases/Incidence(%)
Patients with ≥ 1 SAE event	**25 (50.0)**	**20 (50.0 )**	**45 (50.0 )**
Gastrointestinal disorders	**8 (16.0)**	**9 (22.5)**	**17 (18.8)**
Intestinal obstruction	3(6.0)	1(2.5)	4(4.4)
Lab examinations	**7(14.0)**	**1(2.5)**	**8(8.9)**
White blood cell count decreased	3(6.0)	0	3(3.3)
Platelet count decreased	3(6.0)	0	3(3.3)
Neutrophil count decreased	3(6.0)	0	3(3.3)
Blood and lymphatic system disorders	**6 (12.0)**	**0**	**6 (6.7)**
Anemia	5 (10.0)	0	5 (5.5)
Infections and infestations	**5 (10.0)**	**6 (15.0)**	**11 (12.2)**
General disorders and administration site conditions	**3(6.0)**	**2 (5.0)**	**5 (5.6)**
Metabolism and nutrition disorders	**3(6.0)**	**4(10.0)**	**7 (7.8)**
Decreased appetite	2(4.0)	3(7.5)	5(5.6)

Compared to the individuals in the control group, the patients in the M701 group had a greater incidence of SAE in terms of decreased blood cells and anemia, which may be related to the activation of T-lymphocytes. However, those SAEs in blood cells were manageable and reversible. No patients discontinued the M701 treatment because of severe adverse reactions. The ASEI in this study was CRS which was frequently reported in TCEs treated patients. CRS occurred in 4% of the M701 group and the CTCAE grades ranged from 1 to 2.

### Pharmacokinetics and immunogenicity

Among the 50 patients who received at least one infusion of M701, 42 had at least one blood or ascites sample collected for pharmacokinetics and immunogenicity analysis. These patients were included in the PK analysis set and immunogenicity analysis set. The M701 concentration was below the lower limit of quantitation in most of the serum and ascites samples. The highest serum concentration of M701 was found on day 18, and the arithmetic mean was 6.53 ng/mL. The highest concentration of M701 in ascites was found on day 18, and the arithmetic mean was 26.5 ng/mL.

In the serum, the rate of ADA positivity was 64.3%, which was caused by the M701 treatment, whereas in the ascites, it was 45.2%. As the number of administrations increased, the titer of ADA increased, both in the serum and ascites. However, the ADA titer in ascites was considerably lower than that in serum, and the ratio was about 0.1–0.3.

## Discussion

MA has been a significant challenge for cancer patients and oncologists for decades. Although new drugs for systemic cancer treatment have emerged in the last two decades, significantly prolonging the survival of cancer patients, the issue of MA has become more prominent for patients with advanced-stage cancer. Different drugs or devices have been tested in many clinical trials, including TNFα, IL-2, chemotherapy, filtered ascites, and catumaxomab, among which only catumaxomab completed a controlled randomized clinical trial and was approved by the EMA for MA treatment in 2009 [2]. Due to certain commercial reasons, Catumaxomab was withdrawn from the European market in 2017. However, after eight years, no new, more effective treatments have emerged on the market. As a result, in 2025, the European Commission re-approved the marketing of catumaxomab in the European Union for the treatment of malignant ascites, based on previous clinical research results.

In this study, M701 was constructed on an asymmetric bispecific antibody platform (YBODY^®^) developed by YZYBIO, Inc., with favorable characteristics concerning pairing efficacy, yield, and stability using the CHO cell expression system. In vitro assays revealed that M701 specifically trafficked CD3-positive cells to EpCAM-positive cells and caused lysis of only tumor cells with moderate-to-high EpCAM expression, indicating that M701 has few side effects on normal tissue. In xenograft tumor-bearing mice, M701 inhibited tumor growth but neither the anti-EpCAM antibody nor the anti-CD3 antibody performed such inhibition. In an immunocompetent C57BL/6 mouse model, a surrogate molecule targeting murine EpCAM and CD3 also prolonged the survival of mice with MA.

To evaluate the safety and efficacy of M701, this phase II trial was conducted on advanced cancer patients with moderate-to-large-volume symptomatic MA who had failed at least two lines of systemic treatment or were platinum-resistant. The patients in the M701 group received an IP infusion of M701 after drainage, whereas the patients in the control group received only drainage. Moreover, both groups received the systemic treatments selected by the investigator based on the Chinese clinical guidelines. The results of the safety evaluation revealed that these combinations were well-tolerated and that local infusion of M701 did not increase the risk to patients. The highest incidence of M701-related SAEs was the abnormal level of all kinds of blood cells, including decreased white blood cells, platelets, and anemia, which may be due to the activation of T lymphocytes. However, those AEs were transient and reversible. No patient in the M701 group discontinued treatment due to abnormal laboratory examinations. Owing to its asymmetric structure and human-mouse chimerism, M701 has a moderate CD3 affinity and low immunogenicity, which indicates that M701 has the innate characteristics of low incidence of CRS and human anti-mouse antibody (HAMA) reaction. Therefore, the IP infusion of M701 was performed more than 10 times without causing strong CRS or HAMA. Additionally, hepatic toxicity, which is a common ADR caused by catumaxomab, was not observed in M701-treated patients [43–45]. Moreover, repeated M701 IP infusions did not cause prominent abdominal compartment separation, which is frequently reported in patients who have undergone IP chemotherapy [46, 47].

Regarding effectiveness in controlling MA, the M701 group showed an encouraging effect, with a median PuFS of 75 days, which was better than that of the control group (25 days) and the historical data of a pivotal trial of catumaxomab (46 days) [44]. The extra benefit may come from systemic treatment since the control group in this study had a longer PuFS than the historical data of the control group in the catumaxomab pivotal study (11 days) [44]or the lower grade and incidence of CRS and relative adverse drug reactions. The patients in the control group in this study had more drainage than those in the catumaxomab pivotal study (only once); this fact may also have contributed to the prolonged PuFS in the control group. Systemic chemotherapy or antiangiogenic drugs may also weaken hematopoietic function, which may decrease the effect of T-cell-engaging antibodies and increase the risk of M701 infusion. Therefore, we could only conclude that M701 infusion significantly prolonged the puncture/drainage interval with regular systemic treatments.

According to the analysis of PuFS in the subpopulation, some subpopulations, including female patients and patients who previously underwent chemotherapy or paracentesis, received significant benefits.

As a local treatment, M701 infusion is unlikely to improve OS because of the low dose of M701 (400 µg) and limited distribution in peripheral blood. No objective response was found during this phase II study in either the M701 or the control group (data not shown). However, the M701 group showed a trend toward longer OS in the FAS population and most subpopulations. Gastrointestinal cancer patients have longer OS than ovarian cancer patients, which is consistent with the results of pivotal trials of catumaxomab in MA patients. The increase in OS may be due to improvements in physical status or willingness of patients to continue systemic treatment despite the heavy burden of MA. The latter is likely a more important influencing factor.

Since M701 kills tumor cells in the peritoneal cavity by recruiting and activating T lymphocytes, we hypothesized that patients with higher baseline lymphocyte counts may receive greater benefits from M701 treatment. The results of the study revealed that patients with ≥ 13% RLC in peripheral blood had greater PuFS benefit compared to the FAS population (125 days vs. 75 days). These patients also showed significantly longer OS (152 days vs. 76 days). More than 40% of patients in this subgroup survived for six months, whereas most MA patients typically survive for only 2–3 months [2, 4]. This trend was also observed in the pivotal trial of catumaxomab as well [48].

In the phase II study of M701 in MA patients, the participants in both groups received the systemic treatment recommended by the Chinese clinical guidelines for gastrointestinal cancer patients who had failed two lines of chemotherapy and for ovarian cancer patients who were resistant to platinum-based chemotherapy. Systemic treatments may affect PuFS and OS, however, considering that the allocation of tumor types and the number of previous treatment lines in the two groups were balanced, the bias caused by systemic therapies should be minimal.

Moreover, there are still certain limitations in this study, including: (1) the sample size is relatively small, and the degree of benefit for patients with different types of tumors has not yet been determined; (2) the Quality of Life (QoL) data at different time points were not effectively collected. Therefore, further validation of the clinical value of M701 is needed in larger and more well-designed clinical studies, with sufficient sample size and well collected QoL data. Patient-Reported Outcome (PRO) are strong indicators of improvement in patients’ quality of life.

Given its promising efficacy and safety profile, a multiple-center pivotal stage III clinical research is ongoing in China to evaluate the efficacy and safety of M701. Additional clinical studies are planned involving patients outside of China The importance and benefits of repeated M701 infusions or rechallenge following MA intolerance require further evaluation in these clinical trials.

## Supplementary Information

Below is the link to the electronic supplementary material.

Supplementary Material 1.

## Data Availability

The datasets used and/or analyzed during the current study are available from the corresponding author on reasonable request.

## Abbreviations

MA: Malignant Ascites
PuFS: Puncture-free survival
OS: Overall Survival
SAE: Serious adverse event
TEAE: Treatment emergent adverse event
AESI: Adverse event of special interest
EpCAM: Epithelial cell adhesion molecule
TCEs: T-cell engagers
CD3: Cluster of differentiation 3
CRS: Cytokine release syndrome
TNF-α: Tumor necrosis factor-alpha
IFN-γ: Interferon-gamma
bsAb: Bispecific antibody
CIKs: Cytokine-induced killer cells
ECOG: Eastern Cooperative Oncology Group
RLC: Relative lymphocyte count
IP: Intraperitoneal
PFS: Progression-free Survival
CTCAE: Common Terminology Criteria for Adverse Events
HR: Hazard ratio
ADA: Anti-drug antibody
Mw: Molecular weight
KIH: Knobs-into-hole
FAS: Full analysis set
HAMA: Human anti-mouse antibody
