# Supplementary material for: Development and clinical trial of M701, an Anti-EpCAM × Anti-CD3 bispecific antibody: a targeted intraperitoneal therapy for malignant ascites stemming from advanced solid tumors
Source: Exp Hematol Oncol. 2025 Nov 22;14:136. doi: 10.1186/s40164-025-00727-3 (PMC12661848; doi:10.1186/s40164-025-00727-3)
Supplement: Supplementary file 1 — Supplementary Material 1. [file 40164_2025_727_MOESM1_ESM.docx]

**Appendix 1. Likert Four-point Scale**

**Likert Four-point Scale**

| **Study site** |  | | | |
| --- | --- | --- | --- | --- |
| **Subject No.** |  | | | |
| **Primary tumor** |  | | | |
| **Scoring date** |  | | | |
| **Scored by** |  | | | |
| **Symptoms and signs** | **Degree** | | | |
|  | **None**  **(0 point)** | **Mild**  **(1 point)** | **Moderate (2 points)** | **Severe**  **(3 points)** |
| **Anorexia** |  |  |  |  |
| **Nausea** |  |  |  |  |
| **Early satiety** |  |  |  |  |
| **Vomiting** |  |  |  |  |
| **Abdominal pain** |  |  |  |  |
| **Abdominal swelling** |  |  |  |  |
| **Dyspnea** |  |  |  |  |
| **Fatigue** |  |  |  |  |
| **Swollen ankles** |  |  |  |  |
| **Heartburn** |  |  |  |  |
| **Shifting dullness*** |  |  |  |  |
| **Fluid thrill*** |  |  |  |  |
| **Bulging flanks*** |  |  |  |  |
| **Total score** |  | | | |

*:If the result of the shifting dullness test is positive, record 1 point; If the result of the fluid thrill test is positive, record 2 points; If the result of the bulging flanks test is positive, record 1 point.

Note: This scale was cited from “The trifunctional antibody catumaxomab for the treatment of malignant ascites due to epithelial cancer: Results of a prospective randomized phase II/III trial. Int J Cancer 2010, 127(9):2209-2221”
